# *Estrogen Receptor Beta (ERβ)* May Act as Mediator in Apoptotic Induction of Grape Seed Extract (GSE)

**DOI:** 10.31557/APJCP.2019.20.12.3729

**Published:** 2019

**Authors:** Zahra Abroodi, Nayereh Sajedi, Medi Nikbakht, Mitra Soleimani

**Affiliations:** *Department of anatomical sciences, Isfahan University of Medical Sciences, Isfahan, Iran. *

**Keywords:** GSE, MCF7 breast cancer line, IC_50_, ERβ- caspase-3

## Abstract

**Background:**

Grape seed extract is a complex mixture of polyphenols. Its anti-tumor effects have been reported by several studies. Estrogen receptors (ERs) are commonly considered as important markers for breast cancer. The present study aimed to evaluate the apoptotic effects of GSE on MCF7 breast cancer cells and assessed the expression of *ERβ* during treatment of cells with GSE.

**Material and Methods:**

The half maximal inhibitory concentration (IC_50_) of GSE in MCF7 breast cancer cells were calculated by treating cells with serial dilution of GSE for 48 hours and cell viability evaluated using MTT assay. Then cells assigned to three groups: control (no treatment), DMSO (cells treated with 0.05% of DMSO) and GSE group (cells treated with of GSE for 48 hours). The apoptosis assay was performed by detecting Annexin V protein by flow cytometry. The gene expression of *ERβ* and *caspase-3* was evaluated by Real-Time PCR.

**Results:**

Cells in GSE group treated with GSE IC_50_ concentration for 48 hours. Annexin V staining assay, represented early apoptosis detected by flow cytometry analysis showed significantly higher expression (p<0.01) than control and DMSO groups. Moreover, results of Real-Time PCR showed a significant expression in* ERβ* and *caspase-3* genes in GSE group compared to control and DMSO groups (Fold change = 2.3 and 3.5, respectively).

**Conclusion:**

GSE may induce apoptosis in MCF7 human breast cancer cells by activation of *ERβ* gene.

## Introduction

Breast cancer is the second cause of cancer-related death in females. One in eight women in the USA will be diagnosed with breast cancer over the period of her life. It allocates 23% of the cancer cases and 14% of the cancer deaths worldwide (Power et al., 2018). Researches have showed that among breast cancer survivors the women with healthy life style and a diet high in fruits and vegetables, dietary fiber and low saturated fat, the risk of recurrence reduced by 31% (Duncan, 2004). Dietary polyphenolic substances have been reported to have anti-carcinogenic and anti-mutagenic functions (Zhou et al., 2016). Grape seed extract(GSE) is a nutritious compound with a lot of activities such as anti-oxidant, anti-inflammatory, anti-microbial and anti-aging activities due to high content of polyphenolic agents including galic acid, catechin, epicatechin, epigallo-catechin, Quercetin, Kaempferol, Naringenin, Ferulic acid, Procyanidin and proanthocyanidin (Kaur et al., 2009). Topical application of GSE polyphenolic fraction (GSP), reported to have significant effects on the reduction of tumor volume and tumor multiplicity in mice model of skin carcinogenesis (Xia et al., 2010). It has a chemo-preventive potential against different kinds of cancer such as colorectal, prostate, lung, gastric adenocarcinoma and breast (Katiyar and Athar, 2013). It has been proved that proanthocyanidin induces apoptosis and cell cycle arrest; and reduces cell proliferation in carcinogenic cells (Roy et al.2005). Michigan Cancer Foundation (MCF-7), is a commonly used breast cancer cell line. It was isolated the first time from the pleural effusion of a 69-year-old woman with metastatic breast cancer in 1973. These cells are estrogen and progesterone receptor positive cells and belongs to luminal A molecular subtypes (Comşa et al., 2015). Recently specific molecular markers have been used as targets for diagnosis and therapy of each kind of breast tumor (Inoue and Fry, 2016). Estrogen receptors are the most important of these markers. ERs are members of the nuclear receptor (NR) superfamily. They mediate the impacts of the estrogen hormone (Zhang et al., 2014). Estrogen regulates growth, homeostasis and differentiation in eukaryotic cells but it can increase the risk of breast and endometrial cancer too (Gu et al., 2014). Mitogenic functions of estrogen in etiology of breast cancer is well-documented (Duong et al., 2006). Human estrogen receptors have the same structure as shared by other members of steroid receptor family (Sommer and Fuqua, 2001). Two nuclear estrogen receptor (ER) proteins, ERα and ERβ, exert biological actions of estrogens and belong to a large conserved superfamily of nuclear receptors (Duong et al., 2006). *ERβ* is believed to have a tumor suppressor function and has anti-proliferative and anti-carcinogenic effects while ERα increases carcinogenesis (Pettersson and Gustafsson, 2001). *ERβ* may control proliferation induced by ERα (Lu and Katzenellenbogen, 2017). *ERβ* expression decreases in many breast tumors (Lazennec et al., 2001).* Caspase-3* is a kind of most widely studied executive caspase (O’Donovan et al., 2003) and has a crucial role in the process of apoptosis (Tyas et al., 2000). It is the main player of both apoptotic pathways, the pathway initiated by caspase 8, and mitochondrial pathway, mediates by caspase-9 (O’Donovan et al., 2003). Caspase-3 is produced as an inactive 32-kDa proenzyme, which is cleaved at an aspartate residue to yield a 12-kDa and a 17-kDa subunit. Two 12-kDa and two 17-kDa subunits combine to form the active caspase-3 enzyme (Pu et al., 2017). In the present study we aimed to use MCF7 cell line to assess potential apoptosis effects of GSE on breast cancer cells. 

## Materials and Methods

*GSE*

Proanthocyanidin grape seed extract were purchased from (inc. Sigma, USA), and was dissolved in dimethyl sulfoxide (DMSO) as a 200 mg/ml stock solution. The final concentration of DMSO in the culture medium during GSE treatments did not exceed 0.1% (v/v), and therefore, the same concentration of DMSO were used in vehicle control dish.

*Cell Culture*

Human breast carcinoma cell line MCF7 were purchased from Pasteur Institute, Iran. Cells were seeded in T75 flasks at 1×10^6^ cells/flask in low glucose Dulbecco’s modified Eagle’s medium (DMEM) / F12 containing 10% fetal bovine serum (FBS), 2 mM glutamine, 0.01 mg/ml insulin and 1% penicillin/streptomycin mix at 37^o^C in 5% CO_2_ incubator. The medium renewal performed 2 times per week, cells passaged weekly at a sub-cultivation ratio of 1:3 (Comşa et al., 2015).

*IC*_50_ *and MTT assay*

Determining a 50% effect (IC_50_) for chemical compounds is an informative indicator of cytotoxicity/anti-proliferative. To determining the concentration of GSE that inhibited cell proliferation in MCF7 cells by 50% (IC_50_), as described previously (Yen et al., 2015)., 5 x 10^3^ cells per well were seeded in a 96-well microtiter plates and after reaching to 60-70% confluence, cells were treated either with different concentration of GSE, serial dilutions dissolved in solvent DMSO 0.1% (v/v) (200, 175, 150, 125, 100, 75, 50, 25 µg/ml) for 48 hours; or only treated with DMSO, for 48 hours. The final DMSO concentrations for vehicle control and GSE groups were 0.05%. Four hours before the end of incubation, 100 µl of MTT (prepared as stock solution at 10 mg/ml in PBS), at a final concentration of 0.5 mg/ml, were added to each well. The MTT is based on the ability of individual cells to convert the tetrazolium salt 3-(4, 5-dimethylthiazol-2-yl)-2, 5-diphenyl-tetrazolium bromide (MTT) to its water-insoluble formazan derivative by dehydrogenase enzyme in viable mitochondria in healthy cells. MTT formazan metabolites are visualized as purple intracellular granules and crystals under a light microscope (Florento et al., 2012). At the end of the incubation, the medium was aspirated and replaced with 100μL DMSO to dissolve the formazan crystals. The plates were gently shaken for 20 min in the dark to ensure maximal dissolution of formazan crystals. Reading of plates were done at 580 nm Aliquots of supernatant were transferred into a new 96-well plate and read at 580 nm with scanning spectrophotometer. The optical density of the formazan produced by untreated cells was considered as representing 100% viability. The percentage of cytotoxicity was defined as the relative reduction of the optical density (OD), which correlated to the amount of viable cells in relation to cell control (100%). The cell viability was plotted in a graph and the IC_50_ was calculated to define the optimum dosage of GSE treating MCF7 cells.

*Calculation of IC*_50_

To determine IC_50_, a dose response graph prepared by plotting viability against of GSE concentrations using regression analysis in SPSS software. Based on this graph, the concentration equals to100 µg/ml was defined as GSE IC_50_.

*Treatment of MCF7 cells with IC*_50_ *dosage of GSE*

The MCF7 cells assigned into three groups. Control group, DMSO (vehicle) group and GSE group. Control group received no treatment, DMSO group received 0.05% DMSO and GSE group received 100 µg/ml GSE for 48 hours. At the end of 48th hours, cells were washed with PBS, trypsinized and collected to evaluate by Flow cytometry and Q-PCR.

*Apoptosis detection by flow Cytometry*

One of the earlier events of apoptosis includes translocation of membrane phosphatidylserine (PS) from the inner side of the plasma membrane to the surface. Annexin V, a Ca^2+^-dependent phospholipid-binding protein, has high affinity for PS, and fluorochrome-labeled Annexin V can be used for the detection of exposed PS using flow cytometry. To detect Annexin V in apoptotic cells, Annexin V-Propidium Iodide (PI) double staining method performed using the annexin V-FITC apoptosis detection kit (BD Biosciences, San Diego, USA) as described previously (Usman et al., 2018). Briefly, 1 x 10^5^ MCF7 cells/mL in three groups (Control, DMSO, GSE) were washed with cold PBS, trypsinized and centrifuged at 1,000 rpm; the cell pellet was rewashed with PBS and re-suspended in 100 mL of 1X binding buffer (1 x 10^6^ cells/mL). Then, annexin V–FITC and PI, 5 mL each was added to the cell suspension, and the cells were gently vortexed. Then, the cells were incubated for 20 min at room temperature (25^o^C) in the dark. Then, the samples were diluted by adding 400 mL 1X binding buffer. Annexin-V/ PI fluorescence was studied on BD FACSCalibur. Samples data were analyzed by the Cell Quest Pro software (BD Biosciences).

*Quantitative Real-Time PCR*

The gene expression of *ERβ* and *caspase-3* in MCF7 cells in all groups were evaluated by Real-Time PCR. Total RNA was extracted using the Blood RNA kit (1

Thermo Fisher Scientific Inc., USA), and reverse transcription-PCR was performed routinely with the PrimeScript real-time-PCR Kit (Takara Bio Inc., Shiga, Japan). Diluted cDNA (5ul) was analyzed in triplicate by real-time PCR in an iCycler iQ Detection System (Bio-Rad, Hercules, California, USA) using SYBR Green Universal PCR Master Mix, based on the manufacturer’s recommendations. 

Each sample was normalized on its GAPDH mRNA content. Comparative quantitative method was used according to the formula of 2(DDCT) to calculate the relative fold change of mRNA expression. The primers used for the amplification of the entire *CASP-3* and *ERβ* coding regions were reported previously (Wang et al., 2016) as forward primer, 5´-AAAGGATCCTTA ATAAAGGTATCCATGGAGAACACT-3´ (corresponding to -15 to +12 of human CASP-3 mRNA); and reverse primer, 5´-AAA GAATTCTTAGTGAT AAAAATAGAGTT CTTTTGTGAG -3´ (+834 to +805 of human CASP-3 mRNA) for *Caspase-3* (Wang et al., 2016); forward 5′-CCCTGCTGTG

ATGAATTACAG −3′ and reverse 5′-TCGGTTCCCACT

AACCTTCC-3′ (ER beta); and forward 5′-CCCACTCCTCCACCTT

TGAC-3′ and reverse 5′-TGTTGCTGTAGCCAAATTC

GTT-3′ (GAPDH).

*Statistical analysis*

To determine statistical significance among all groups, one-way analysis of variance (ANOVA) was performed using SPSS software version 25. Tukey used as post hoc test. Data have been presented as the mean ± standard deviation and significance was accepted at a level of P < 0.05.

## Results

*Cell viability and MTT assay*

To assess the potential harmful effects of GSE on MCF7 cells, cell viability was determined by MTT assay. A significant concentration-dependent was observed particularly at higher dosage of GSE (200, 175, 150, 125, 100, 75, 50, 25 µg/ml) for 24 hrs.

*Dose response curve and IC*_50_

**Figure 1:**
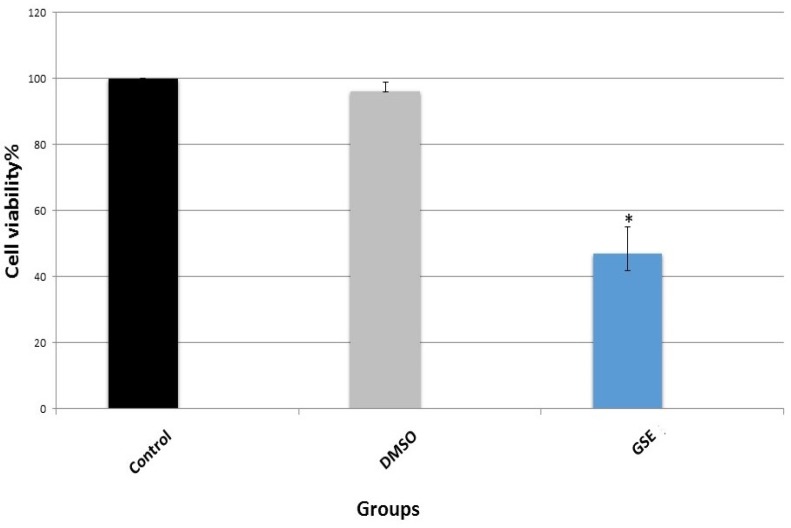
Cell Viability as Evaluated by MTT Assay, GSE Compared to Control and DMCO Groups. Asterisks depict significant difference compared to other groups

The half maximal effective dose (IC_50_) of GSE deduced from dose-response curve generated by plotting percentage of viable cell (% cell viability) against of GSE concentration. Based on the curve depicted on Figure 1, IC_50_ defined as 100 µg/ml.

*Apoptosis detection by flow Cytometry*

**Figure 2:**
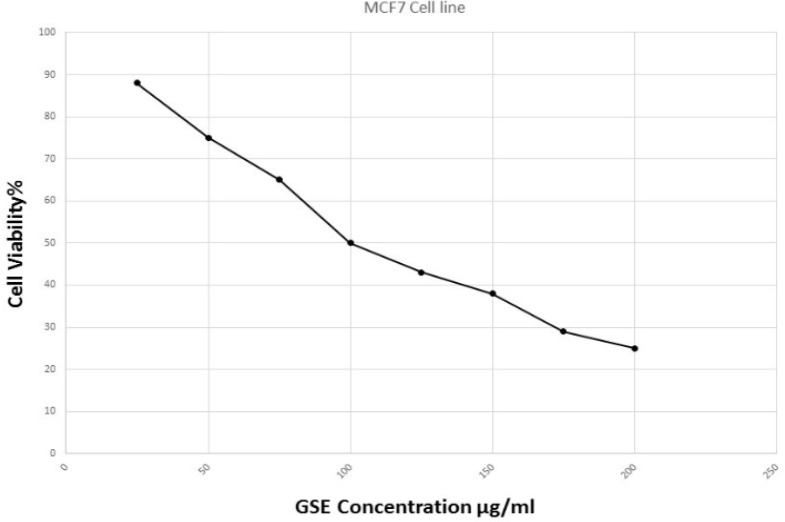
Dose-response curve of GSE on MCF7 breast cancer cells. Cells were treated with 200, 175, 150, 125, 100, 75, 50, 25 μg/ml of GSE for 48 hrs. The Cell viability was evaluated by MTT assay and the concentration point in the curve that adjusted to 50% of the cell viability considered as IC_50_ for GSE

The flow cytometry results showed that GSE induced apoptosis in MCF7 cells (Figure 2). Dot plots in the first quadrant (upper left) represents necrotic cells. The second quadrant (upper right) represents late apoptotic cells that could be simultaneously stained with Annexin V-FITC /PI. The third quadrant (lower left) represents normal viable cells were not stained with Annexin V-FITC and propidium iodide (PI), and finally the fourth quadrant (lower right) represents cells that only stained by Annexin V-FITC for early apoptosis. The early apoptotic rate of the GSE group (56%) was significantly (P < 0.01) higher than the early apoptotic rate of the DMSO (vehicle) group (11%) and Control group (7%). However, the early apoptotic rate of the DMSO (vehicle) was not statistically different than the early apoptotic rate of control group.

*Gene Expression*

Real-Time PCR results showed that the expression of caspase-3, an apoptosis marker, in GSE group was significantly (P < 0.01) more than the expression of this gene in DMSO group and control group as calculated as relative quantification (fold change=3.5). Moreover, the expression of ERβ in GSE group was significantly more than DMSO and control group (Fold change= 2.3).

## Discussion

In the present study, we investigated the apoptotic effects of Grape Seed Extract (GSE) on breast cancer MCF7 line. To determine the potential harmful dosage of GSE on MCF7 cells, we used MTT assay to calculate IC_50_, the concentration of GSE that induce 50% of apoptosis on MCF7 cells. Cells divided into three groups, Control (MCF7 cells in normal condition with no treatment), DMSO or vehicle (MCF7 cells treated with DMSO 0.05%) and GSE (MCF7 cells treated with 100 µg/ml GSE). GSE is a nutraceutical agent that is consumed as a dietary and health supplement. Antioxidant content of GSE is in the form of proanthocyanidins and is greater than vitamin C and E (Katiyar and Athar, 2013). Antitumor-promoting activity of GSE has been suggested by several reports (Wen et al., 2008). It also has been showed that GSE hinder the growth of cells of colon cancer cell lines both in vitro and in tumor xenografts in nude mice (Kaur et al., 2009). It is reported that GSE induces apoptosis in human prostate cancer cell line(Gao et al., 2009). In one study has been suggested that GSE has some effects as synergy with doxorubicin in inhibiting the growth of estrogen-receptor-expressing MCF-7 cells as well as estrogen-receptor negative MDA-MB468 cells (Sharma et al., 2004). To assess apoptotic effects of GSE in breast cancer we used fluorochrome-labeled Annexin V assay by flowcytometry. Annexin V is a Ca2+-dependent phospholipid-binding protein and has high affinity for PS, and fluorochrome-labeled Annexin V can be used for detection of exposed PS. The obtained result of this study showed that in GSE group the expression of Annexin V protein was significantly more than its expression in DMSO and control groups. This may approve apoptotic effects of GSE extract on breast cancer MCF7 cells. Caspase-3 is a kind of most widely studied executive caspase (O’Donovan et al., 2003) and has a crucial role in the process of apoptosis (Tyas et al., 2000). It is the main player of both apoptotic pathways, the pathway initiated by caspase-8, and mitochondrial pathway, mediates by caspase-9 (O’Donovan et al., 2003). Therefore, *caspase-3* is an important marker of apoptosis. Real-Time PCR used in the present study to evaluate the expression of caspase-3 following treatment of MCF7 breast cancer cells with GSE. The results showed significant increase of apoptosis in GSE group compared to control and DMSO groups. This reiterate the apoptotic impacts of GSE on MCF7 cells. Estrogen receptors (ERα and/or ERβ) mediate functions of estrogens. While ERα is believed to have carcinogenic effects, ERβ exhibits an inhibitory action on ERα-mediated gene expression and, in many instances, opposes the actions of ERα (Al-Bader et al., 2011). It also reported that ERβ was unable to regulate c-myc proto-oncogene expression(Liao and Dickson, 2000). These all support anticancerogenic effects of ERβ. To evaluate influences of GSE on expression of *ERβ*, the expression of* ERβ* was evaluated by real-time PCR. We observed that following 48 hours of treatment with GSE, the expression of *ERβ* increased significantly than control and DMSO groups. However, the expression of *ERβ* was not significantly different in control and DMSO groups. This may suggest that apoptotic properties of GSE may mediate by ERβ.

In conclusion, treatment of breast cancer MCF7 cells with GSE induced apoptosis in these cells. GSE may exert its apoptotic influences by mediation of ERβ.

**Figure 3:**
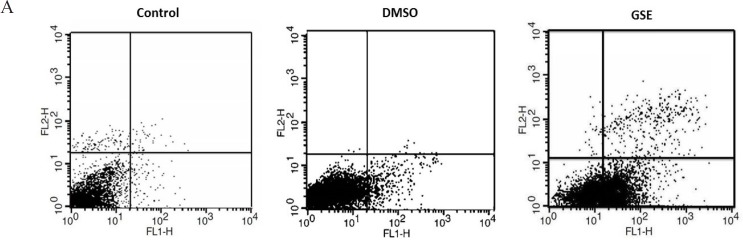
A. GSE induced Annexin V/PI-based apoptosis in MCF7 cells. Cells were treated with indicated GSE concentrations (0–200 μg/ml) for 48 h. (A) Histograms of representative Annexin V-FITC profile in GSE-treated MCF7 cells. (B) Quantitative analysis for the apoptotic cells (%). Apoptosis was counted at the intensity of right gated region in (A). Data, mean ± SD (n = 3).

**Figure 3:**
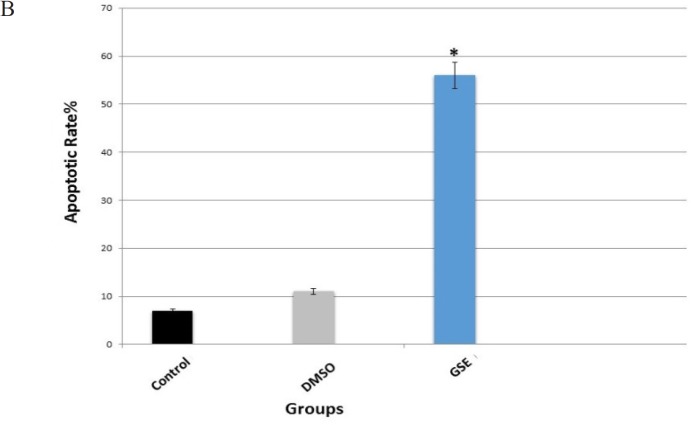
B. Flow Cytometric Analysis of Annexin V-FITC/PI Assay of MCF-7 Cells Treated with Indicated Concentrations of GSE. Representative dot plots show the viable cells (%), early apoptosis (%), late apoptosis (%) and necrotic cells (%) (A) Control, (B) DMSO and GSE. (D) Bar diagrams are showing the percentage of apoptosis observed by flow cytometric analysis of MCF-7 cells in three groups. Data presented in ±SE. * Significant (p < 0.05) compared with controls

**Figure 4:**
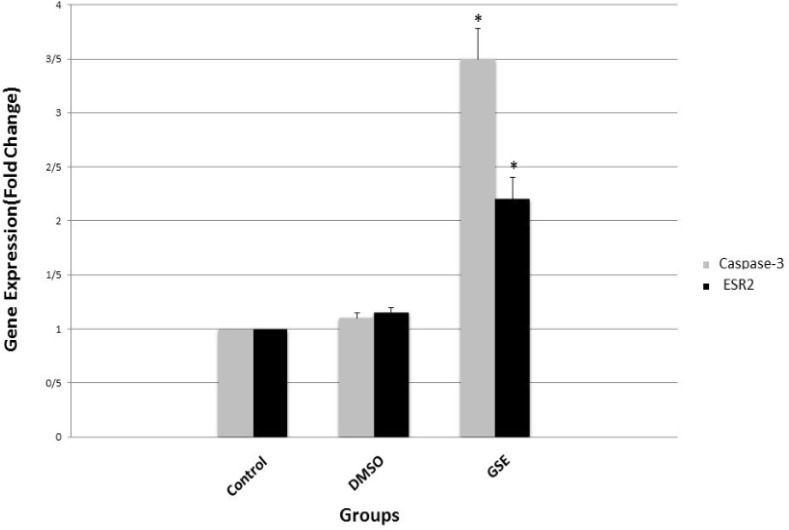
Relative Expression Analysis of *ERβ* and *Caspase-3 *Genes in MCF-7 Cells after 48 Hours of Treatment with GSE Compared to the Control and DMSO Groups by Real-Time PCR. Data presented in ±SE. * Significant (p < 0.01) compared with controls
